# The role of legumes in the sustainable intensification of African smallholder agriculture: Lessons learnt and challenges for the future

**DOI:** 10.1016/j.agee.2019.106583

**Published:** 2019-11-15

**Authors:** B. Vanlauwe, M. Hungria, F. Kanampiu, K.E. Giller

**Affiliations:** a International Institute of Tropical Agriculture, PO Box 30772, Nairobi, 00100, Kenya; b Embrapa Soja, PO Box 231, CEP 86001-970, Londrina, PR, Brazil; c Plant Production Systems, Wageningen University, PO Box 430, AK, Wageningen, the Netherlands

**Keywords:** Best-fit technologies, Biological N_2_ fixation, Legume agronomy, Legume-rhizobium symbiosis, Rhizobium inoculants, Rotational benefits

## Abstract

Grain legumes play a key role in smallholder farming systems in sub-Saharan Africa (SSA), in relation to food and nutrition security and income generation. Moreover, because of their N_2_-fixation capacity, such legumes can also have a positive influence on soil fertility. Notwithstanding many decades of research on the agronomy of grain legumes, their N_2_-fixation capacity, and their contribution to overall system productivity, several issues remain to be resolved to realize fully the benefits of grain legumes. In this paper we highlight major lessons learnt and expose key knowledge gaps in relation to grain legumes and their contributions to farming system productivity. The symbiosis between legumes and rhizobia forms the basis for its benefits and biological N_2_-fixation (BNF) relies as much on the legume genotype as on the rhizobial strains. As such, breeding grain legumes for BNF deserves considerably more attention. Even promiscuous varieties usually respond to inoculation, and as African soils contain a huge pool of unexploited biodiversity with potential to contribute elite rhizobial strains, strain selection should go hand-in-hand with legume breeding for N_2_-fixation. Although inoculated strains can outcompete indigenous strains, our understanding of what constitutes a good competitor is rudimentary, as well as which factors affect the persistence of inoculated rhizobia, which in its turn determines whether a farmer needs to re-inoculate each and every season. Although it is commonly assumed that indigenous rhizobia are better adapted to local conditions than elite strains used in inoculants, there is little evidence that this is the case. The problems of delivering inoculants to smallholders through poorly-developed supply chains in Africa necessitates inoculants based on sterile carriers with long shelf life. Other factors critical for a well-functioning symbiosis are also central to the overall productivity of grain legumes. Good agronomic practices, including the use of phosphorus (P)-containing fertilizer, improve legume yields though responses to inputs are usually very variable. In some situations, a considerable proportion of soils show no response of legumes to applied inputs, often referred to as non-responsive soils. Understanding the causes underlying this phenomenon is limited and hinders the uptake of legume agronomy practices. Grain legumes also contribute to the productivity of farming systems, although such effects are commonly greater in rotational than in intercropping systems. While most cropping systems allow for the integration of legumes, intercropped legumes provide only marginal benefits to associated crops. Important rotational benefits have been shown for most grain legumes though those with the highest N accumulation and lowest N harvest index appear to demonstrate higher residual benefits. N balance estimates often results in contradictory observations, mostly caused by the lack of understanding of belowground contributions of legumes to the N balance. Lastly, the ultimate condition for increased uptake of grain legumes by smallholder farmers lies in the understanding of how legume technologies and management practices can be tailored to the enormous diversity of agroecologies, farming systems, and smallholder farms in SSA. In conclusion, while research on grain legumes has revealed a number of important insights that will guide realization of the full potential of such legumes to the sustainable intensification of smallholder farming systems in SSA, many research challenges remain to be addressed to realize the full potential of BNF in these systems.

## Highlights

• More effort is needed to integrated biological nitrogen fixation potential in breeding schemes under N-limiting soil conditions.
• In combination with strain selection efforts, it is likely that the amount of N fixed by grain legumes can substantially increase.
• A better understanding of the major factors affecting grain yield is required for improving best-bet agronomic practices and associated risks.
• An important challenge is to unravel the limiting factors in non-responsive soils.
• Information on intercropping benefits to associated crops or N balances in rotations is insufficient to judge the sustainability of such systems.

## 1 Introduction

Smallholder agriculture in sub-Saharan Africa (SSA) needs to intensify either because expanding agricultural land is no longer an option for densely populated areas, or to ensure that natural ecosystems, such as the forest in the Congo Basin, are preserved. Even in areas where land expansion still occurs, intensification of agricultural production is needed to keep pace with an ever-growing population. The discourse on intensification is currently framed as ‘Sustainable Intensification’ (SI), and commonly encompasses three dimensions: (i) increased productivity; (ii) maintenance of ecosystem services; and (iii) increased resilience to shocks (e.g., Pretty et al., 2011; Vanlauwe et al., 2014).

Integrated Soil Fertility Management (ISFM) is central to sustainable intensification in Africa given that poor soil fertility is the primary production constraint (Vanlauwe et al., 2015). Legumes play a key role in ISFM due to their ability to fix atmospheric N_2_ in symbiosis with rhizobia; they supply organic resources and can counteract other constraints by enhancing fertilizer uptake, suppression of weeds, among other benefits (e.g., Sanginga et al., 2003). The link between legumes, SI, and improved farmer livelihoods is easily made since N in legumes is fixed with a substantially lower greenhouse gas footprint than fertilizer N (Sá et al., 2017). Furthermore, legumes provide organic inputs with positive impacts on soil chemical, physical and biological properties, thus improving crop yields. When an opportunity arose to focus on enhancing the role of N_2_ fixation by legumes in Africa, a decision was made to focus on grain legumes (Giller et al., 2013). Although green manures and legume trees can fix larger amounts of N_2_ from the air, smallholder farmers priorities the production of grain legumes because they provide immediate benefits as nutritious food and for sale (Giller et al., 2013). The resulting project “N2Africa - Putting Nitrogen Fixation to Work for Smallholder farmers in Africa” started in 2009 (www.n2africa.org).

The major grain legumes in Africa are cowpea (*Vigna unguiculata* (L.) Walp.), chickpea (*Cicer arietinum* L.), groundnut (*Arachis hypogaea* L.), common bean (*Phaseolus vulgaris* L.), faba bean (*Vicia faba* L.), pigeonpea (*Cajanus cajan* (L.) Millsp.), and soybean (*Glycine max* (L.) Merr.). Of these, chickpea, faba bean and, to a lesser extent, common bean are mainly found in highland regions (above 1000 m). Related to their growth habits and capacity to fix N_2_, legumes contribute to the SI of farming systems in varying degrees, from likely minimal (e.g., short-cycle bush beans) to substantial (e.g., dual purpose soybean, pigeonpea). Though a relatively cheap practice with clear potential to increase yield substantially, the use of rhizobial inoculants on legumes in SSA was limited to a few countries until recently (e.g., Zimbabwe, South Africa) and mostly on soybean (Mpepereki et al., 2000; Chianu et al., 2011).

**Fig. 1:**
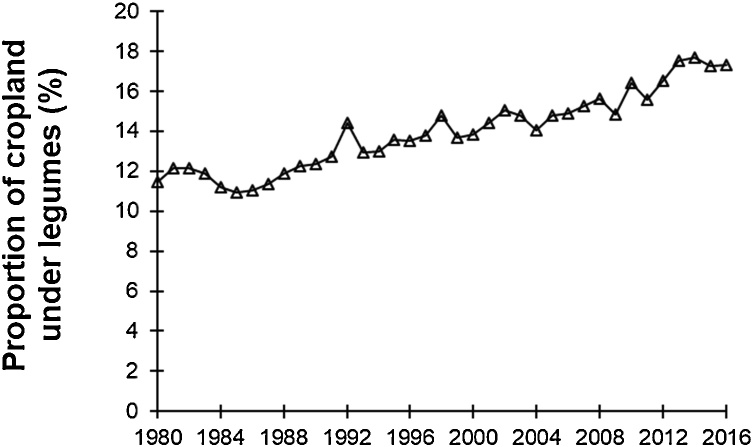
Proportion of cropland in sub-Saharan Africa under legumes for the period 1980 - 2016. Legumes considered included, following the FAO terminology: ‘Bambara beans’; ‘Beans, dry’; ‘Broad beans, horse beans, dry’; ‘Chick peas’; ‘Cow peas, dry’; ‘Groundnuts, with shell’; ‘Peas, dry’; ‘Pigeon peas’; and ‘Soybeans’. Source: www.fao.org.

Notwithstanding all above potential benefits, the area under legumes is relatively small in most farming systems in SSA (Mtambanengwe and Mapfumo, 2009; Fig. 1), with the most prominent legumes being grain legumes. Lots of earlier efforts to introduce other types of legumes in smallholder farming systems have largely failed because of their lack of immediate benefits (e.g., tree legumes in alley cropping systems or herbaceous legumes in legume-cereal rotations) (Adesina et al., 1999; Manyong et al., 1999; Douthwaite et al., 2002). Grain legumes have the benefit of providing immediate returns to farmers in terms of grains that can be either consumed in the household, or sold; in addition, crop residues can be used as high quality livestock feed. Grain legumes can also be integrated in nearly all farming systems, as monocrops in rotation with a cereal (e.g., soybean, groundnut) (Sanginga et al., 2002), as intercrops between root crops, banana (*Musa* spp.), or cereals (e.g., cowpea, beans, pigeonpea) (Pypers et al., 2011), or as ‘doubled up’ legumes between other legumes (e.g., pigeonpea – groundnut (Snapp et al., 2010).

**Fig. 2:**
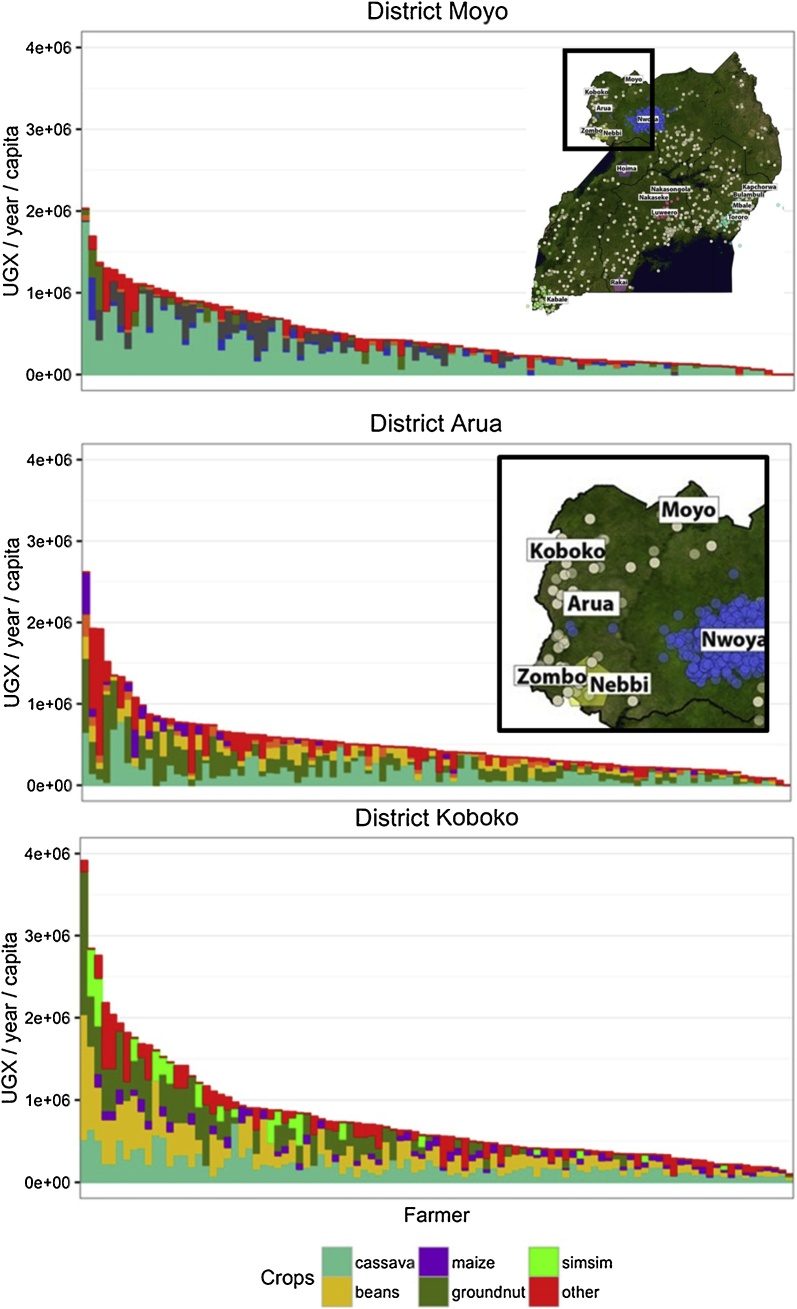
Value of production (UGX refers to Uganda Shilling) per capita per year for various production units for a set of households at three sites in Northern Uganda (Moyo, Arua, Koboko), noting that each horizontal bar represents one household. ‘Simsim’ is most often referred to as sesame. Data extracted from the paper by Kansiime et al. (2018).

For these major reasons, the N2Africa project focuses on intensification of cowpea, soybean, groundnut, bush and climbing beans, faba bean, and chickpea. If grain legumes are produced mainly for home consumption, then one would not expect large areas under such crops. For instance, a family of seven, requiring a total of c. 60 kg protein per year, could obtain this from 165 kg of soybean or 290 kg of common beans, equivalent to 33 or 58 m^2^ of legumes, if the yields are of 2 and 1 t ha^−1^, respectively. Where grain legume production exceeds these areas, it is most often related to profitable access to markets. Kansiime et al. (2018), for instance, noted that farmers in North-West Uganda would only increase the area under groundnut or beans in the proximity of markets paying acceptable prices for such products (Fig. 2), and in most countries, e.g., Mozambique, the demand for soybean grows in association with poultry production (e.g., Smart and Hanlon, 2014).

**Fig. 3:**
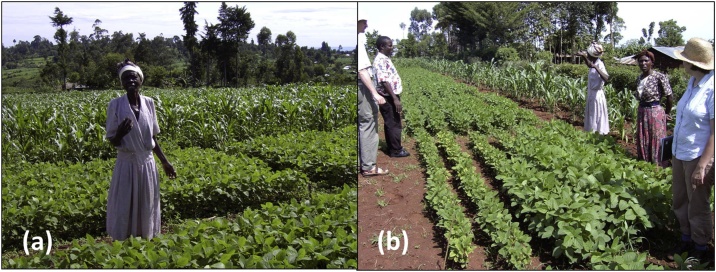
Soybean growth on a relatively fertile plot near the homestead (a) and on a relatively degraded site on an outfield (b) in Western Kenya. Note the likely symptoms of K deficiency in the insert in photograph (b). Photographs: B Vanlauwe.

Smallholder farming communities and farming systems are heterogeneous in many respects, including access to resources, production objectives, cropping diversity, and soil fertility conditions (Fig. 3). The major food security crops in specific farming systems vary with agro-ecological conditions such as altitude and rainfall conditions, often included in the description of farming systems, e.g., cassava (*Manihot esculenta* Crantz), maize (*Zea mays* L.), or banana (*Musa* spp.) -based systems, and nearly all farming systems contain a locally ‘preferred’ grain legume. Within farming communities, better off, commercial production-oriented households commonly produce more market-oriented grain legumes, such as soybean in Northern Nigeria, while poorer, subsistence-oriented farmers will rather produce smaller areas of grain legumes for household consumption (e.g., beans in Eastern DR Congo). In relation to soil fertility conditions, an appreciable proportion of soils in densely populated smallholder areas is often labeled as non-responsive, indicating that crop grown on such soils do not respond to the application of commonly-available fertilizers (Vanlauwe et al., 2010). Because of all this complexity and diversity, Ojiem et al. (2006) proposed the concept of the socio-ecological niche for the integration of legumes in smallholder farming systems. Based on this concept, the N2Africa project was designed around a central hypothesis that BNF by legumes, grain and biomass yield depends on:

BNF ∼ (G_L_ × G_R_) × E×M

where G_L_ = the legume genotype; G_R_ = the genotype(s) of rhizobia nodulating the legume; E = the environment, including climate (temperature, rainfall, day length, etc., to encompass the length of the growing season) and soils (acidity, aluminum toxicity, limiting nutrients, etc.); M = management, including agronomic practices (rhizobial inoculation, use of mineral fertilizers) (Giller et al., 2013). Further, the legume technologies need to be tailored to the local circumstances, needs, resources and aspirations of the farmers (Ojiem et al., 2006; Vanlauwe et al., 2010).

Recently, a special virtual issue of Agriculture, Ecosystems, and Environment on ‘The role of nitrogen (N) fixation in African smallholder Agriculture’ (Giller et al., 2018) was published. The main objectives of this paper are: (i) to highlight major cross-cutting issues emerging from papers in above special issue and (iii) to highlight key knowledge gaps and identify research priorities. The paper first focuses on the legume-rhizobium symbiosis, then covers more general aspects of legume agronomy and productivity, and ends with highlighting interactions between grain legumes and overall farming system characteristics.

## 2 Legume-rhizobium symbiosis

The main approaches to ensure the establishment of an effective legume-rhizobium symbiosis in Africa include: (i) to breed legume genotypes with increased BNF efficacy with elite strains; (ii) to select grain legumes, or legume genotypes that are sufficiently promiscuous to nodulate effectively with the indigenous population of rhizobia present in the soil; and (iii) to inoculate with effective rhizobia strains.

### 2.1 Biological N_2_ fixation relies as much on the legume genotype as on the rhizobial strains

Differences between genotypes in BNF capacity have been reported for a variety of legumes (Dwivedi et al., 2015). However, there are legumes such as common bean with several reports of low contributions from BNF (Graham, 1981; Herridge et al., 2008; Peoples et al., 2009). One main hypothesis is that such failure results not from the rhizobial strain, but from the lack of plant breeding for high BNF capacity. Therefore, host plant breeding is mandatory to improve BNF, especially if inoculation with elite strains is expected to increase yield. Efforts towards introducing BNF as a main property to be considered in plant breeding could have profound impacts on symbiotic performance. However, nowadays there is no strong program aiming to breed legumes for BNF. Intriguingly, projects with less achievable probability of success, such as the introduction of bacterial N_2_-fixation genes in non-legumes, have received more financial support and interest. It is now time to invest more in legume breeding programs aiming to directly enhance the genetic potential for BNF contribution.

The importance of the environment, including temperature, soil moisture, and soil fertility, each of which has potentially high impacts on the BNF performance by legumes (Hungria and Vargas, 2000; Dwivedi et al., 2015). Therefore, genotypes unable to deal with abiotic and biotic limitations cannot express their BNF potential. For some legumes, such as common bean, besides jeopardizing nodule functioning, soil properties such as acidity can affect competitiveness of rhizobial species (*e.g.* Streit et al., 1992; Anyango et al., 1998), requiring thorough analysis of the strain to be delivered in commercial inoculants at each site. In Africa, results indicate that emphasis should be given to P nutrition (e.g. Didagbé et al., 2014) and, indeed, impressive yield increases have been attributed to the combined application of P and inoculant (Ronner et al., 2016).

Breeding of grain legumes in Africa has recently focused on drought tolerance, among other biotic and abiotic constraints. The development of improved varieties can be seen as a technology pipeline, where new traits are included and combined with other important characteristics such as grain type, taste, nutrition, and cookability. Breeding for genotypes with high BNF potential needs to be combined as an extra important trait and this can be readily achieved through selection under N-limiting soil conditions with rhizobium inoculation. Such conditions can be readily achieved in breeding nurseries through rotation with cereal crops without addition of N fertilizers. Selection for other soil constraints such as soil acidity are more difficult and need careful selection of locations for screening based on representative infertile soils. Little attention has been placed on breeding grain legumes for BNF during the last two decades, except for the continuation of the breeding of soybean for promiscuity in nodulation at the International Institute of Tropical Agriculture (IITA).

### 2.2 Promiscuous legume varieties usually respond to inoculation

Almost two decades ago, Giller (2001) concluded that grain legumes that could nodulate and fix N_2_ without inoculation were the best options for smallholder farmers due to the perceived problems of delivering rhizobial inoculants. At that time, this conclusion was reached largely because inoculants were not available in the great majority of the countries of SSA. In addition, the reports were of very rare responses to rhizobial inoculation in promiscuous legumes such as cowpea and pigeonpea.

The lack of commercial inoculants in Africa affected mainly the soybean crop, an exotic legume to Africa. Whereas soybean was generally considered to be fairly specific in its rhizobial requirements, greater promiscuity was observed as early as in the 1960s in Zimbabwe, and in the 1970s in Tanzania (Mpepereki et al., 2000; Giller, 2001). An extensive soybean breeding program was then established in the late 1970s at IITA in Nigeria, which had breeding for promiscuity in nodulation as one of its major goals, resulting in the TGx (Tropical *Glycin*e cross) varieties (Pulver et al., 1985). This breeding program has produced well-adapted, promiscuous soybean varieties with prolific growth and N_2_ fixation and good yields, resulting in their adoption by millions of farmers in West Africa. These varieties have also proven to be broadly adapted for smallholder farming conditions in East and southern Africa.

Nevertheless, it was also shown early on that these promiscuous varieties could benefit from rhizobial inoculation (Ranga Rao et al., 1985). Recently several studies have shown that inoculation with the exotic strain USDA 110 of *Bradyrhizobium diazoefficiens*, or with other elite strains can improve yields of promiscuous soybean varieties in Africa (e.g., Chibeba et al., 2018; Rurangwa et al., 2018; van Heerwaarden et al., 2018). However, responses to inoculation are still stronger in non-promiscuous cultivars, as shown in 55 trials across five countries, where the average yield response of specific soybean was higher than that of promiscuous varieties (van Heerwaarden et al., 2018). Promiscuous genotypes thus still represent an interesting approach for farmers that do not have access to inoculants. However, as the commercialization of inoculants advances, yield increases can be obtained with promiscuous genotypes through inoculation of elite strains. Recent results have also demonstrated that promiscuous legumes can respond to inoculation. For example, although cowpea is probably one of most promiscuous legumes studied, strains have been identified within the biodiversity of the Brazilian soils that give consistent inoculation responses in both Brazil (Martins et al., 2003), and Ghana (Boddey et al., 2017; Kyei-Boahen et al., 2017). Inoculation with indigenous strains has also proven to be very effective in increasing yield of promiscuous common bean in Brazil (Hungria et al., 2003).

As African soils are rich in genetic and symbiotic diversity of rhizobia, they are also an important reservoir of elite strains. In the past decade, identification of elite strains has been a priority in several African countries for soybean (e.g., Sanginga et al., 2000; Musiyiwa et al., 2005; Abaidoo et al., 2007; Chibeba et al., 2017), common bean (Kawaka et al., 2014; Mwenda, 2017), chickpea (Tena et al., 2016), and groundnut (Abdullahi, 2016). In addition, the reported high diversity detected in rhizobia nodulating Bambara groundnut (*Vigna subterranea* (L.) Verdc.) (Grönemeyer et al., 2014), groundnut and hyacinth bean (*Lablab purpureus* (L.) Sweet) (Grönemeyer et al., 2014; Abdullahi, 2016) may represent a valuable source for bio-prospecting for superior BNF properties.

The breeding and selection program of IITA has focused on identifying well-adapted, high-yielding soybean varieties that are promiscuous in their nodulation. Selection has been done through breeding without inoculation with rhizobium so that the genotypes nodulate with native rhizobial populations, as described above. A danger of this breeding strategy is that the genetic composition of native rhizobium populations will vary among locations, so that the selected genotypes may not be equally able to nodulate when introduced into new areas. In the long-term, assuming that inoculants will become generally available, breeding for enhanced BNF with known inoculant strains is likely to lead to more stable and higher yields.

It is understood that promiscuous legumes can respond to inoculation and that African soils contain a large number of elite strains. The technology pipeline for all grain legumes needs greater attention to the (G_L_ × G_R_) × E combinations which require breeding programs for improved grain legume varieties together with ongoing rhizobial strain selection programs. At present concerted breeding programs exist only for the major grain legumes such as cowpea and groundnut. As far as we know there are no centres in Africa that conduct routine rhizobial screening. Ideally, legume breeding and strain selection should go hand-in-hand, taking into account the huge diversity of agroecologies within which all of the grain legumes are grown.

### 2.3 Inoculated rhizobium strains can compete with indigenous strains

Competitiveness (defined as the capacity to form large proportion of the legume nodules in the presence of populations of indigenous/naturalized rhizobia) certainly represents the greatest challenge to the introduction of elite strains, and it has often been pointed out as the main factor explaining inconsistent responses to inoculation (e.g., Graham, 1981; Keyser and Cregan, 1987; Thies et al., 1991). Limitations caused by soil rhizobial populations have been described for many legumes, from promiscuous hosts, such as common bean (Graham, 1981), to exotic soybean such as with strains of serogroup USDA 123 in the USA (Keyser and Cregan, 1987).

It is difficult to define the genetic basis of competitiveness, and various studies have indicated correlations with strain attributes such as chemotaxis, motility, production and composition of exopolysaccharides, and antimicrobial compounds, in addition to environmental conditions (Archana, 2010). Conflicting results reported in the literature might be explained by regulation of competitiveness by a pool of genes and not any single one. Unfortunately, probably due to complexity, after a series of studies in the 1980s and 1990s, including the construction of genetically engineered more-competitive strains, e.g., by producing an anti-rhizobial peptide (Triplett, 1990), or by altering indole acetic acid biosynthesis (Kuykendall et al., 1996), interest in the subject cooled. On the other hand, genomic studies are revealing genes and operons that might be implicated in competitiveness, stimulating a resurgence in interest in this topic (Archana, 2010).

Many studies focus on the identification of indigenous rhizobia with the expectation that they will be better adapted to local soil conditions and better able to fix N_2_ with locally-grown legumes. This has been thought mainly based on the premise that indigenous strains are more competitive. Yet evidence to support this assumption is scant – in fact exotic strains commonly used in commercial inoculants have a broad adaptation. For example, *B. diazoefficiens* USDA 110 shows outstanding adaptation and performance in a broad range of soil types and countries, including Africa (e.g., Chibeba et al., 2018; Rurangwa et al., 2018). Similarly, *Rhizobium tropici* CIAT 899, originally isolated in Colombia has proved to be consistently good inoculant strain for common bean in Africa (Rurangwa et al., 2018), and Brazil (Hungria et al., 2003), and exotic strains isolated in Brazil have increased yields of cowpea in Ghana (Boddey et al., 2017; Kyei-Boahen et al., 2017).

From the knowledge obtained in the few past years, with an emphasis on the results of the N2Africa project, it seems feasible to find suitable inoculant strains for all legumes, from those specific for nodulation such as non-promiscuous soybean (e.g., Hungria and Mendes, 2015; Chibeba et al., 2018), to those that nodulate promiscuously, such as promiscuous soybean, common bean and cowpea (e.g., Hungria et al., 2003; Martins et al., 2003; Chibeba et al., 2018). This requires very rigorous strain selection for both higher rates of N_2_-fixation and nodulation competitiveness. The opportunity of improving legume grain yield by inoculation with elite strains is highlighted by van Heerwaarden et al. (2018). The results of a combined analysis of more than 2000 trials with soybean, showed that inoculation increased yields by an average of 115 kg ha^–1^ (or 9.5% increase relative to the uninoculated treatments), with 75% of the fields showing responses between 102 and 172 kg ha^–1^; even more importantly, in 97% of the fields economic improvements accrued.

Our understanding of what determines whether a rhizobial strain is a good competitor against populations of native rhizobia remains rudimentary. Despite the widespread assumption that screening of indigenous rhizobia will lead to better local adaptation and identification of elite strains better than those generally used in inoculants, there is little evidence for this. What determines local adaptation and competition for nodulation among rhizobia is an area that requires a major research effort.

### 2.4 Re-inoculation can be beneficial

Considering that elite strains are available, albeit possibly partially limited in saprophytic capacity and competitiveness, it is important to know which inoculation strategy will most likely be successful in introducing elite strains to agricultural soils. Performance should be investigated on a strain-by-strain basis. In one type of response, the strains perform well in the first year, but does not establish successfully in the soil, requiring re-inoculation, such as has been reported for clover with *R. leguminosarum* bv. trifolii (Roughley et al., 1976) and with soybean strain CPAC 7 (Mendes et al., 2004). Another group of strains requires two to three years to establish in the soil, after which they are highly competitive and difficult to displace, such as those in the serogroup USDA 123 (in the USA, Keyser and Cregan, 1987), SEMIA 566 and CPAC 15 (in Brazil, Mendes et al., 2004; Hungria and Mendes, 2015) and E109 (in Argentina, Brutti et al., 1998). In a third case, re-inoculation every year with the same strain guarantees higher nodule occupancies and yields, as it is often observed for soybean (Hungria and Mendes, 2015) and common bean (Hungria et al., 2003) in South America. Therefore, we conclude that responses to re-inoculation can be positive. The positive results obtained by re-inoculation of elite strains, at least in the tropics, in addition to the high cost of N-fertilizers-mostly imported in these countries, and therefore subject to international prices, and to the low cost of inoculants confirm the feasibility of using inoculants as a cheap and little risk source of N.

Rhizobial inoculants have been produced locally in Zimbabwe since 1967 and used in soybean production for 50 years (Giller et al., 2011). Soybean was produced predominantly on large scale farms and became a popular crop of smallholder farmers in Zimbabwe only over the past two decades. Given the very sandy soils, poor in soil organic matter, found in many of the smallholder farming areas in Zimbabwe it was hypothesized that rhizobial survival in the soils might be problematic, requiring repeated inoculation each year. Indeed, Zengeni et al. (2006) found that rhizobial numbers fell rapidly within two years after inoculation, but could be maintained if the soil was amended with cattle manure. Nevertheless, re-inoculation every season appears to be the most sensible strategy, especially in view of the low cost of application of inoculants, only requiring a marginal increase in legume yield to recover the purchase and application costs.

Further research attention to understanding factors that determine the persistence of inoculated rhizobia is warranted, and this may differ widely among different rhizobial species and strains. Finding strains that establish well and are persistent in soils when their host legume is not cropped will be useful for farmers. On the other hand, if strains are highly competitive and establish well it may be difficult to displace them if more effective inoculants strains are identified. Given the low cost of rhizobium inoculants it is perhaps wise to recommend re-inoculation with every crop until we have a better understanding of persistence in the field. Once a better understanding of the rhizobial persistence is achieved, farmers could then be provided with legume- and soil-specific guidelines as to how many years between sowing the same legume species sufficient populations of rhizobia might be expected to survive following a previously inoculated crop to still provide adequate nodulation.

### 2.5 Delivery of inoculants is critical in smallholder farming conditions

The increasing reports of benefits of inoculation in Africa, mainly generated through the N2Africa project, have increased the commercial interest in inoculant supply. Interestingly, several multinational companies are now investing in biological products, for nutritional and pests and diseases control, alerting that the perception of the feasibility of using microorganisms for profitable agriculture is increasing. However, taking advantage of the microbiological marketing towards agricultural sustainability, a variety of microbial inoculants with no guarantee of agronomic efficiency, or of the identity or quality of the microorganisms has been commercialized (e.g., Herrmann et al., 2015). It is important to consider that after the introduction of “wrong” strains in the soils, they will may persist forever, with negative impacts on plant production.

On the contrary, a broad range of microorganisms and microbial processes have been identified that can be highly beneficial for plant growth. For example, there have been reports of precocious and more abundant nodulation and impressive increases in grain yields of soybean and common bean due to co-inoculation of rhizobia and *Azospirillum brasilense* (e.g., Hungria et al., 2013). In the near future, multipurpose inoculants carrying compatible microorganisms acting influencing different microbial processes could be made available to farmers, bringing important contributions to agriculture sustainability.

Due to the lack of cooled supply chains in sub-Saharan Africa, the shelf life of inoculants that are produced based on pasteurized peat is limited and remaining contaminants rapidly overgrow the rhizobial populations. The shelf life of inoculants produced with sterile peat, e.g., through irradiation, is commonly larger; in addition, it reduces the risk of introducing and disseminating plant, animal and human pathogens (Hungria et al., 2005). Moreover, due to the relatively limited acreages of smallholder soybean farms, the package size of inoculants should be small.

Several results obtained through the N2Africa project and other initiatives have raised farmers’ interest in inoculants. Consequently, strain trials and local production and importation of inoculants is growing exponentially. However, it is most important to establish strong legislation to guarantee that only inoculants with confirmed efficacy and quality are commercialized, and an accompanying independent quality control laboratory.

Although inoculants of excellent quality are now available for purchase in most countries, most are still inoculants produced on non-sterile carriers. The shelf-life of inoculants that contain contaminants is often only a few weeks, which means that they may not contain sufficient effective rhizobia by the time they are applied by farmers. This is particularly problematic when inoculants are sold in small packets, which are much more susceptible to damage on exposure to high temperatures or poor handling. New manufacturing processes that can produce large numbers of small packets of high quality suitable for the smallholder market are required.

## 3 Legume agronomy and productivity

**Fig. 4:**
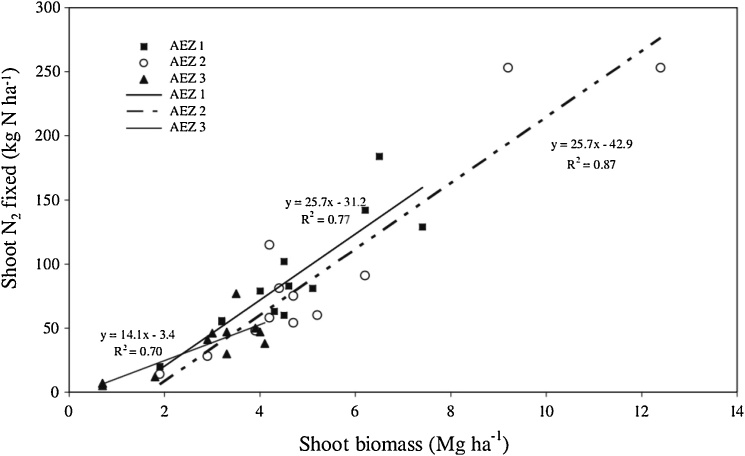
Inputs from N_2_ fixation by legumes are much larger when the legumes grow prolifically as shown across three agroecological zones (AEZs) in Western Kenya (after Ojiem et al., 2014).

Experience across many countries of sub-Saharan Africa shows that grain legumes can achieve excellent yields, e.g., sometimes exceeding 3 t ha^−1^ for soybean, and concomitant large amounts of N_2_-fixation, e.g., in excess of 150 kg ha^−1^. High rates of N_2_-fixation depend on prolific growth of the legume (Fig. 4). As stressed, in addition to tailoring a compatible and effective G_L_ x G_R_ combination to the local environment (E), crop management (M) needs to be optimal for successful yield and N_2_-fixation of grain legumes. In this section, we consider the importance of the ‘management’ (M) component. First, we consider general aspects of crop management and how they influence response to inputs, then we focus on aspects of soil fertility management.

### 3.1 Good agronomic practices positively affect legume productivity

In Malawi, the most popular interventions to enhance yields among farmers were early sowing date, optimal plant population and choice of variety (van Vugt et al., 2017). Preventative spraying of chemical control against Asian rust (*Phakopsora pachyrhizi*) in soybean gave positive economic returns at sites where disease incidence was severe, but a loss of income when the disease did not occur. Farmers ranked interventions that did not require cash investment above rhizobial inoculants and fertilizer even though they recognized the benefits of inputs in enhancing crop yields (van Vugt et al., 2017).

Recent studies have demonstrated a large variability in yield response of grain legumes to inoculation and P in farmers’ fields in sub-Saharan Africa. A key benefit of large-scale projects such as N2Africa that have been implemented over the last ten years is the opportunity they allowed testing of grain legume response to rhizobium inoculation and P in many farmers’ fields. These experiments consisted of simple comparisons of the best local variety of the grain legume with and without inoculation and P and with inoculation and P combined to give four plots in single replicates in each farmer’s field (Ronner et al., 2016). Despite the highly contrasting climates and soils across different countries, surprisingly similar patterns of crop response have been observed. In all cases, there is a very wide variability in yields – often ranging from close to no yield at all to yields close to the genetic potential of the legume variety at around 4 t ha^−1^. The overall mean effects of inoculation and P on grain yields when applied alone were often in the region of an extra 0.5 t ha^−1^ of yield (or 47% relative to the uninoculated treatment without P added). The combined effects of inoculation and P were additive rather than synergistic (whereby the effect of the combined application is larger than the sum of the effects of the individual components), giving a yield increase of 0.8-1.0 t ha^−1^ (Ronner et al., 2016; Wolde-Meskel et al., 2018). Attempts to explain the variability in legume yield and yield response have given disappointing results. Although 40–60% of the soybean yield response in Northern Nigeria was explained by year, farm size, plant establishment, total rainfall and soil pH, this had little predictive value across locations or seasons (Ronner et al., 2016). In the case of climbing beans yields increased with the density and quality (length, stalks or wood) of the stakes used to support them (Franke et al., 2019). Other variables such as farm size also emerged as important (Ronner et al., 2016) – and in some cases such variables were heavily confounded with the experimental location and farmers’ wealth status (Franke et al., 2019). Given that land and labour availability and soil fertility status are often strongly dependent on the relative wealth of the farmers it is not surprising that it is difficult to tease apart the relative importance of different factors in explaining crop yields and yield responses to treatments.

It is unclear what the underlying reasons are for our lack of ability to predict the yield response to inputs. It could be due to insufficiently detailed or accurate information on crop management and environmental variables (soil and climate). Understanding the variability in crop response is key to being able to predict the likelihood of success of the technology for farmers. Such information would also assist in enriching decision support tools that provide more specific recommendations and their associated risks and deserves urgent research attention.

### 3.2 Influence of soil fertility on legume yields

The inherent soil fertility status is key to ensuring good legume yields, as has been demonstrated with a range of grain legumes in Kenya (Ojiem et al., 2007), with groundnut and soybean in Zimbabwe (Zingore et al., 2008a), with climbing bean in Rwanda (Franke et al., 2019) and with cowpea, groundnut and soybean in Ghana (Kermah et al., 2018). In most of these cases the differences in soil fertility could be attributed mainly to past management with organic manures, with fields closer to the homestead tending to be more fertile than those further away (Ojiem et al., 2007; Zingore et al., 2008a; Kermah et al., 2018). The amount of N_2_ fixed by the grain legumes was much larger in more fertile fields where soil conditions are more favourable (Ojiem et al., 2007; Kermah et al., 2018; van Vugt et al., 2018). That said, it has been reported that the application of N-fertilizer or high amounts of mineral N in the soil decrease the contribution from BNF (e.g. Van Kessel and Hartley, 2000; Hungria et al., 2006).

Grain legume growth and production in the tropics is often limited by lack of available P in the soil (Giller and Cadisch, 1995). Grain legumes have relatively sparse root systems in terms of extent and root length density compared to cereal crops. Therefore, P uptake by legumes can be more limiting than with grasses, making the legumes highly dependent on arbuscular mycorrhizal symbiosis. One rare exception might occur with some species of lupins (*Lupinus* spp.), that appear not to associate with mycorrhiza, but more studies are needed to clarify this (Shi et al., 2017). In a proportion of the farmers’ fields, no response to P fertilizer or inoculation was observed - ranging from 8% of the fields with chickpea in Ethiopia (Wolde-Meskel et al., 2018) to more than 30% with soybean in Kenya (Thuita et al., 2018). Fields where no response was observed tended to be those where the yield of the control plots was also very poor – indicating that factors other than available P were limiting crop growth and yield. The P fertilizers used in the experiments were often ‘straight’ P fertilizers such as single superphosphate (SSP) (Ronner et al., 2016), triple superphosphate (TSP) or whichever P fertilizer was locally available, such as diammonium phosphate (DAP) (e.g., Rwanda: Franke et al., 2019). None of these P fertilizers provide a balanced fertilization.

While rhizobial inoculation is an inexpensive technology, rarely costing more than 10 USD ha^−1^ in SSA (or even lower in other continents, e.g., in South America, inoculants rarely cost more than 3 USD ha^−1^), P fertilizer tends to be four to five-fold more expensive on a hectare basis. The additional yield obtained due to inoculation when applied together means that the cost of the P fertilizer is readily covered and the combination is much more economically attractive (Ronner et al., 2016; Thuita et al., 2018; Wolde-Meskel et al., 2018). Both the likelihood of breaking even and the overall return on investment are increased markedly when both rhizobial inoculants and P fertilizers are applied together.

In Kenya a fertilizer blend (Sympal) that included magnesium (Mg) and zinc (Zn) as well as calcium (Ca) gave more consistent and stronger improvements in yield than a P fertilizer than contained Ca (Minjingu) (Thuita et al., 2018). In some of the multivariate analyses, variables that were selected with a statistically significant contribution to explaining variability in crop yield were soil chemical variables such as pH or exchangeable Mg, or physical variables such as sand content (Ronner et al., 2016), but in no cases were these the most important variables.

Diagnosis of micronutrient deficiencies is notoriously problematic. Soil chemical tests do not reflect the bioavailability of micronutrients for plant uptake and are thus very poor indicators of micronutrient deficiency. Micronutrient deficiencies are most likely to occur in soils that are either very acid, and therefore present multiple problems for plant growth such as deficiencies in cations such as Ca and Mg and toxicity of aluminium (Al) and/or manganese (Mn) (Sanchez and Salinas, 1981; Brodrick et al., 1992), or in very sandy soils that are poor in soil organic matter and also exhibit deficiencies of multiple nutrients (Rebafka et al., 1993; Zingore et al., 2008b).

While it has been demonstrated beyond doubt that legumes require the addition of available P once soil available P levels are below a specific threshold, e.g., in the range of 10–15 mg kg^−1^ Olsen-P, there is less clarity on (i) which other nutrients are required to increase legume yields, (ii) what would be thresholds for these nutrients, below which responses are expected, (iii) how such limitations are related to past management of the legume field, and (iv) how such limitations are geospatially expressed. These research questions require specific attention if the legume yield gap is to be narrowed for large areas in SSA partly through the formulation and deployment of legume-specific fertilizers.

### 3.3 Non-responsiveness of legumes to fertilizer

Non-responsive soils are soils on which crops do not show any meaningful response to the application of a specific fertilizer formulation (Vanlauwe et al., 2010). Also for legumes, a minimal proportion of plots in multi-locational trials show little to no response to applied fertilizer. For instance, Thuita et al. (2018) observed a lack of response to Minjingu and Sympal fertilizer applied to soybean in about 40% and 30%, respectively of the fields. Wolde-Meskel et al. (2018) observed that for about 20% of the fields, the response of chickpea to P application was less than 100 kg ha^−1^. Van Vugt et al. (2018) found that for about 20% of the fields, soybean yields that had received inoculant and fertilizer were similar to those in the no-input control plots).

The reasons why a poorly-yielding crop does not respond to additional standard nutrients are several and related to soil, management, pest/disease and/or weather factors. For example, there could be localized waterlogging or drought, crop management (e.g., weeding) may have been inadequate, or the soils may have physical or chemical sub-soil constraints restricting root growth. In many cases however, we suspect that other nutrients are limiting crop response. In pot trials with 22 non-responsive soils from four countries, it was observed that apart from P deficiency, which was ubiquitous, the nutrients most frequently found to be limiting for plant growth were potassium (K) and to a lesser extent Mg (Keino et al., 2015; Lenoir, 2014; Seitz, 2013).

Many more detailed studies are needed to identify local nutrient deficiencies and needs. The frequency and magnitude of grain legume response to K and sulphur (S) appear sufficient to warrant their ubiquitous use – these nutrients should be added to all basal legume fertilizers. The need to include other macro (e.g., Mg) and micronutrients (e.g., B, Zn) is not clear. Convincing evidence of micronutrient deficiencies is rare and where they are observed they tend to be sporadic and not widespread across the landscape. Soil analysis is simply not sensitive enough to diagnose micronutrient deficiencies. Moreover, the availability of many different nutrients is influenced by soil acidity and alkalinity and legumes differ in their relative tolerance to acid or alkaline soils (Brockwell et al., 1991). This means that widespread crop response experiments are needed in areas where micronutrient deficiencies are suspected. Collecting information on responses from multi-locational trials with detailed data collection schemes in relation to soil, weather, management and pest/disease expression could also assist in creating a better understanding on which of those are actually mostly responsible for non-responsiveness of legumes crops to fertilizer.

## 4 Contribution of grain legumes to farming systems

Legumes are well known for their multiple contributions to cropping systems which culminate in improved soil health in line with sustainability principles (Foyer et al., 2016). Due to the versatility of legumes in terms of growth types and duration, legumes are present in various spatial and temporal niches of nearly all cropping systems in SSA, thus affecting the potential contributions to those systems.

### 4.1 Intercropping legumes

Intercropping offers large opportunities for intensification of a number of cropping systems. In crops that are slow to establish such as cassava, grain legumes can be intercropped during the first rainy season. A modified crop arrangement with cassava planted at 2 m × 0.5 m increased legume (common bean) production without negatively affecting cassava yield; in addition, it resulted in higher legume production during the first season and permits a second common bean intercrop, resulting in an added economic benefit of almost USD 1000 ha^−1^ (Pypers et al., 2011). Agronomic measures, plant densities, crop arrangements and relative planting times can greatly increase productivity of the system. Hernández et al. (1999), for example, found higher land equivalent ratios in a cassava–common beans intercrop sown three weeks after planting of cassava in a system with alternate rows of both crops, in comparison with mixed rows of both crops planted simultaneously. Dapaah et al. (2003) showed that short stature, small and less-branching cassava varieties have the least negative effects on a legume intercrop.

A series of studies comparing different intercropping arrangements of grain legumes with maize indicate that within row intercropping is more attractive in both yields and economic returns than strip or alternate row intercrops (Rusinamhodzi et al., 2012; Falconnier et al., 2016; Kermah et al., 2017). Within row intercrops have the advantage of retaining the planting density of maize – which is often considered by farmers as the main, food security crop – while providing the additional legume yield. Kermah et al. (2017) found that the land equivalent ratios (LERs) of grain legume-maize intercrops tended to be greater in less fertile fields and in poorer rainfall environments although total productivity decreased. In Mali, a ‘within-row’ additive maize-cowpea intercrop gave an LER of 1.47 without any maize yield penalty and an extra 1.4 t ha^−1^ of cowpea fodder (Falconnier et al., 2016). Climbing varieties of *P. vulgaris* are successfully intercropped at high elevations in South America with maize providing physical support to the beans. Climbing Vigna spp. have also been found to be successful as intercrops in maize in SE Asia (Rerkasem et al., 1988). Experience in Africa suggests that the climbing varieties are too vigorous and tend to smother maize.

Whilst the total amount of N_2_-fixed per hectare tends to be less in intercrops that in sole crops, due to the decreased productivity of the legume, the proportion of N fixed is increased (Giller, 2001). For example, Kermah et al. (2017) found that the proportion of N fixed tended to be greater in the intercrops on soils of poor fertility (55–94%) than in fertile soils (23–85%), although the total amounts of N_2_-fixed were less (15–123 kg N ha^−1^ compared with 16–145 kg N ha^−1^). This increased dependence on N_2_-fixation of intercropped legumes results from the decreased availability of soil N due to more competitive and efficient uptake of soil N by the companion (cereal) crop (Giller, 2001).

The amount of N transferred from a legume to associated crops is a subject of considerable controversy. It varies depending on conditions that impact legume N-fixation; legume species, symbiotic performance, and agronomic factors (Chalk, 1997; Giller, 2001; Adu-Gyamfi et al., 2007; Makoi et al., 2009). While some reports indicated that N is transferred directly to intercropped non-legumes, others have found no benefit of N transfer to the associated crops. In Congo, a study by Mandimba (1995) concluded that the N contribution of groundnut to the growth of maize in intercropping systems was equivalent to the application of 96 kg of N ha^−1^ at a plant population ratio density of one maize plant to four groundnut plants. In western Nigeria, Eaglesham et al. (1981) observed that in a cowpea-maize intercrop, 24.9% of N_2_-fixed by cowpea was transferred to maize. In Tanzania, using the ^15^N isotope dilution method, Vesterager et al. (2008) reported that cowpea fixed 36 kg N ha^-1^ when intercropped with maize as estimated around peak biomass production. Some 19% of the fixed N (8 kg N ha^−1^) was indicated to be transferred to maize but, as highlighted by the authors, the isotope dilution method suffers from a number of problems that call such estimates into question (e.g., Chalk et al., 2014).

While intercropping legumes provides interesting agronomic benefits, the issue of how much (fixed) legume-N is transferred to the associated non-legume crop remains an important research issue, especially in view of the N balance and sustainability dimensions of such systems. This question applies to the direct transfer of N from the legume during its growth as well as to the N benefits to the associated crop after the legume is harvested, e.g., through earlier litter fall or remaining roots and nodules.

### 4.2 Rotations with legumes and inputs from N2-fixation for subsequent crops

A common observation has been the increase in grain yields of cereal crops planted after the legume and which has been attributed in part to the legumes’ contribution of N requirement of cereal crops. These contributions have been grouped under major titles of fixed-N and non-N or cropping pattern effects but these effects have rarely been separated (Yusuf et al., 2009; Carsky et al., 2002. The ‘non-N’ or ‘break-crop’ effects include benefits to organic matter improvement, soil structure, water availability, improved P mobilization and reduced pressure from pests and diseases. The fixed-N effects have been reported to range from 124 to 279 kg ha^−1^ for grain legumes (Yusuf et al., 2009), while non-N effects can range from 193 and 600 kg ha^-1^ (Giller, 2001; Yusuf et al., 2009).

**Fig. 5:**
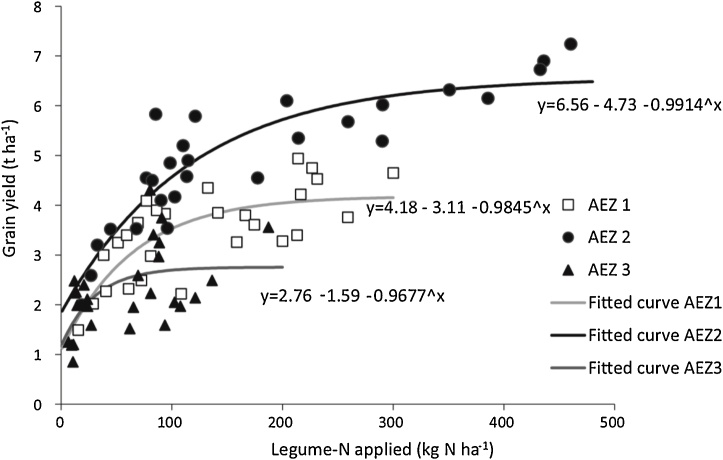
Grain yields of maize grown in rotation are clearly related to the amount of N returned to the soil across three agroecological zones (AEZs) in Western Kenya (after Ojiem et al., 2014).

There is strong evidence that greater inputs from N_2_-fixation from legumes lead to greater residual benefits for subsequent crops (e.g., Fig. 5; Zingore et al., 2008a; Ojiem et al., 2014). In a meta-analysis of 44 studies conducted in Africa, the mean effect of growing maize after a grain legume was roughly a 0.5 t ha^−1^ increase in yield compared with maize after maize (Franke et al., 2018). All grain legumes gave significant residual benefits for cereals, and overall groundnut and soybean gave stronger yield increases than cowpea. Mean yields of maize grown after soybean in Malawi were 3.5 t ha^−1^ compared with 2.5 t ha^−1^ in maize after maize (van Vugt et al., 2018). In Rwanda, residual benefits of common bean and soybean to maize were observed with maize yields which ranged from 0.8 t ha^−1^ in control plots to 6.5 t ha^−1^ in treatments previously inoculated with P and manure added for maize grown after common bean and from 1.9 t ha^−1^ in control plots to 5.3 t ha^−1^ for maize grown after soybean (Rurangwa et al., 2018).

Yields of maize grown after the promiscuous soybean varieties were observed as generally double those of maize grown after the non-promiscuous soybean varieties (Sanginga et al., 2004; Mpepereki et al., 2000). These soybean varieties produced higher quantities of biomass, and likely made highest contributions from N_2_ fixation but which also contributed to an enriched soil organic carbon especially when haulm and other residues were not exported. For an equivalent grain yield of 2 t ha^−1^, promiscuous soybean varieties produced 150 kg N ha^-1^ in stover compared with new varieties that had only 40 kg N ha^-1^ in their stover. However, benefits of cowpea rotation are sometimes higher than expected based on the N content of the cowpea crop alone. Reasons for this include substantial root biomass and N, substantial N-sparing by the legume, and other benefits such as reduction in *Striga hermonthica*, or pests and often diseases, and possibly access to sparingly soluble P (Thierfelder et al., 2012; Franke et al., 2018). There is dearth of information on the contribution of climbing beans to associated rotation or associated crop components. Increases in yield of maize associated with climbing beans ranged from 17 to 38% depending on the variety and date of planting of the bean and had consequences for bean yields where increasing days of planting after maize led to decreasing increase in the bean yields (Gebeyehu et al., 2006; Abebe et al., 2017).

In principle, legume genotypes that derive a high proportion of their N from fixation and have a relatively low N harvest index should be best for rotation systems. Most grain legumes can obtain between 50 and 80% of their total N requirements through biological fixation, but some, like faba bean or soybean can fix up to 90%. Even when legumes grow well, the contribution to soil fertility depends on the amount of N_2_ fixed in relation to the amount removed from the system in the crop harvest, reflected in the N-harvest index (Giller and Cadisch, 1995). For example, creeping varieties of groundnut and cowpea tend to have low harvest indices for N. In contrast, high yielding varieties of soybean usually have high N-harvest indices and often are net removers of soil N (Toomsan et al., 1995).

Our understanding of the impacts of legume rotations and intercrops on the overall N balance of the cropping system and on soil organic matter is weak. We see large increases in yield of cereals when rotated with legumes which are often maintained even when substantial N fertilizer is added (similar absolute increases, smaller proportional increases). The conundrum is that the net N balance of grain legumes is often negative (i.e. more soil N appears to be removed in the harvested crop than the amount of fixed N that is left in the soil). This raises the question: Are the calculations of N balance wrong? About one third of the total N_2_ fixed by legumes is assumed to remains in the roots (Herridge, 2011; Peoples et al., 2009), and may be either used by the following crop or transformed into soil organic matter. Considering this, then older studies that indicate negative N balances when legumes are grown (e.g., Giller and Cadisch, 1995; Wani et al., 1995) are incorrect. Therefore, the negative N balance is not real because it was based solely on above-ground production data. Assuming that one third of the fixed N ends up in the root systems, then the N balance after growing a grain legume is likely to be positive, which explains the many reports of increased yield of subsequent crops (Peoples et al., 2009; Hungria and Mendes, 2015).

Alternative explanations for the net benefits of legumes are the ‘sparing’ of N (less N removal than a cereal), or the non-N effects as indicated above (Giller, 2001). It seems likely that advances in our methods for studying the N cycle are required to resolve these questions. There is also a clear need for more detailed mechanistic studies to assess the occurrence and relevance of non-N effects of grain legumes, particularly in relation to common pests and diseases in cereals. Studies are needed to unravel the non-N effects when integrating grain legumes in farming systems. In general, the nature of this contribution varies widely due to the varying methods used to measure the quantity of N_2_ fixed by the legume and to the variable prevailing environmental factors especially those that greatly influence the productivity of the legume-based cropping systems. This obviously creates a knowledge gap in our understanding of mechanisms underlying the principles of N and non-N benefits to the associated or following cereal crop.

### 4.3 From ‘best bet’ technologies to ‘best fits’

A key focus of our research has been the question ‘How do we tailor legume technologies and management practices to the enormous diversity of agroecologies, farming systems and smallholder farms in sub-Saharan Africa?’ Within the N2Africa project we used the concept of the socio-ecological niche (Ojiem et al., 2006) in the design and planning of deployment of technologies. We employed three strategies to evaluate the factors that influenced the suitability of technologies, namely (i) comparing different practices and technologies; (ii) evaluating the performance of technologies in different contexts; and (iii) monitoring those factors that were difficult to predict (Farrow et al., 2019). Thus tailoring technologies to be suitable for a diversity of farmers across locations is a multi-dimensional and multi-level puzzle.

At a larger scale, the climate determines whether conditions are suitable for the different grain legumes – in particular the temperature, rainfall and length of growing season. The clearest examples among the grain legumes grown in SSA are the ‘cool season’ legumes such as chickpea and faba bean which are most common at high elevations. At a more local level, choice of variety of the grain legumes match the length of growing season becomes important given the wide variation in growth duration within each crop. No clear patterns in terms of preferential performance of the different grain legumes across soil fertility gradients has been observed (Ojiem et al., 2007; Kermah et al., 2018).

Two approaches proved essential in understanding the tailoring of legume technologies for the huge diversity of farmers: the tools of farming systems analysis were essential to gain a deeper understanding of the contexts at different scales (Descheemaeker et al., 2016), and participatory approaches were key in understanding farmers preferences and constraints (van Vugt et al., 2017; Falconnier et al., 2017; Ronner et al., 2018). Perhaps inevitably, wealthier farmers benefit most from yield-enhancing interventions in absolute terms (Franke et al., 2019; van Vugt et al., 2018), as was expected based on *ex ante* analyses (Franke et al., 2014). Wealthier farmers are more likely to use inputs (Farrow et al., 2019). Yet some approaches to intensification are within the reach of even the poorer farmers, such as use of new varieties. In Malawi, farmers prioritized early planting and increased plant density when ranking technologies (van Vugt et al., 2017), although the poorest farmers are often delayed in their own fields by working for others for food at a critical period of the ‘hunger season’ (Kamanga et al., 2014). Poorer farmers cultivated climbing beans more often than wealthier farmers but used fewer of the practices demonstrated (Ronner et al., 2018). Participatory experiments with alternatives to staking for climbing beans produced disappointing results as methods using strings proved to be ‘more tiresome’ (Ronner et al., 2018). An important observation was that farmers use of various management practices varied from season to season (Ronner et al., 2018). Particularly input use depends on the availability of the inputs for purchase and the farmers’ ability to pay, such that practices that did not require extra cash outlay tended to be more popular (van Vugt et al., 2017; Ronner et al., 2018). Participatory co-learning cycles over several seasons proved to be a powerful method to develop tailored options for farmers (Falconnier et al., 2017).

The challenge of ‘best-fit’ scaling remains. Farrow et al. (2019) provide a framework for stratification of contextual factors to create ‘adoption domains’. By choosing sites carefully across these domains we hope to be able to tease out which key, higher-level factors facilitate scaling of technologies. Factors at local levels such as farmers’ resource endowment require different approaches. Through participatory learning closely with farmers in few villages, a ‘basket of options’ can be created which is broad enough to offer practices within the reach of all households. Such research will inevitably need to be repeated in new areas where the farming systems are not well-studied.

## 5 Conclusions

Over the past decades, substantial progress has been made in understanding grain legume agronomy, the symbiosis between those legumes and rhizobia populations, the benefits of BNF to farming systems, and the spatial and temporal integration of legumes in these systems. That said, important knowledge gaps prevent the formulation of recommendations that would further enhance the contributions of legumes to farming systems in SSA.

In relation to the legume-rhizobium symbiosis, more effort is needed to integrated BNF potential in breeding schemes under N-limiting soil conditions. In combination with strain selection efforts, including the use of novel molecular tools and approaches, it is likely that the amount of N_2_ fixed by grain legumes can substantially increase, especially in those legumes that are known to fix relatively low amounts of N. The need to re-inoculate needs to be better understood as well as the most appropriate rhizobium delivery systems, adapted to the supply chain conditions in SSA.

In relation to legume agronomy and productivity, a better understanding of the major factors affecting grain yield is required for improving best-bet agronomic practices, associated with assessments of risk of non-performance. While it has been demonstrated that P is a critical nutrient for optimal BNF and legume growth, information on the geo-spatially expressed limitations of other nutrients, is required. Another important challenge is to investigate the limiting factors in soils that do not allow crops to respond to fertilizers or inoculants, referred to as non-responsive soils.

In relation to contributions of grain legumes to farming systems, information on intercropping benefits to associated crops or N balances in rotational systems is insufficient to judge the N requirements and sustainability of such systems. Both systems have shown important agronomic benefits in terms of overall yields of associated or subsequent crops but the specific mechanisms underlying such added benefits are less well understood. Obviously, such systems need to consider the diverse agro-ecological conditions and the diversity of smallholder farmers towards the identification of ‘best-fit’ solutions.

Investments in resolving above research gaps will ensure that grain legumes do deliver on their potential benefits to farming systems and thus to smallholder livelihoods in SSA. Globally, the benefits should also be extended to environmental impacts, as a result of reduced use of N fertilizer, increased use efficiency of fertilizer, and enhanced inputs of C into the soil.
